# Optimising planned patient transportation: A qualitative descriptive study of current practices in KwaZulu-Natal, South Africa

**DOI:** 10.4102/safp.v68i1.6362

**Published:** 2026-09-23

**Authors:** Bhekithemba R. Cebekhulu, Nombeko Mshunqane, Tshikani L. Khoza

**Affiliations:** 1 Department of Emergency Medical Care and Rescue, Faculty of Health Science, Durban University of Technology, Durban, South Africa; 2 Discipline of Physiotherapy, School of Health Science, University of KwaZulu-Natal, Durban, South Africa

**Keywords:** planned patient transportation, referral systems, healthcare access, service delivery, patient outcomes, South Africa

## Abstract

**Background:**

Planned patient transportation is a key component of healthcare provision in KwaZulu-Natal. However, persistent inefficiencies in the transportation system have been associated with suboptimal health outcomes among patients. Understanding current planned patient transportation practices is therefore imperative to guide improvements in the Emergency Medical Care referral system. This study aimed to describe current planned patient transportation practices in KwaZulu-Natal, South Africa.

**Methods:**

A descriptive qualitative design was employed. Semi-structured interviews were conducted with 11 purposively selected Emergency Medical Services managers, and five focus group discussions were conducted with 35 pre-hospital emergency care providers recruited using maximum variation purposive sampling. Data were transcribed verbatim and analysed thematically using NVivo. Ethical approval was obtained from the relevant ethics committees.

**Results:**

Findings revealed four superordinate themes related to enablers of effective planned patient transportation: (1) digital transformation and operational innovations; (2) stakeholder collaboration and community engagement; (3) decentralised healthcare delivery; and (4) policy coherence.

**Conclusion:**

Planned patient transportation is essential for healthcare access and continuity of care but remains constrained by coordination, resource, organisational and policy challenges. Strengthening referral systems, stakeholder communication, policy standardisation, digital health systems and resource allocation may improve transportation effectiveness and support patient-centred, equitable primary healthcare delivery in KwaZulu-Natal and similar settings.

**Contribution:**

The findings provide insights to guide planned patient transportation service improvement through system strengthening and process enhancement.

## Introduction

Planned patient transportation (PPT) services are arranged transport for non-emergency patients between health facilities within a referral system, such as the transport of patients for specialist care and the repatriation of patients recovering from illness.^1^ In KwaZulu-Natal (KZN), PPT services were introduced within Emergency Medical Services (EMS) in 1984 and rolled out province-wide in 2002, playing a vital role in supporting patients with chronic conditions who require access to ongoing specialist services and treatment.^1,2^ As a key healthcare system component, PPT services enable continuity of care by facilitating patient access to appropriate levels of care along the referral pathway.^3^

Appropriate patient transport systems play a critical role in improving healthcare access, bridging geographic and socioeconomic inequalities, and facilitating equitable health service delivery.^4,5,6^ In South Africa (SA), where health services are provided through a tiered system, effective transport systems are crucial to strengthening referral pathways and timely access to healthcare. This aligns with the National Department of Health’s vision of improving population health outcomes through improved access to health services.^7^ Yet, despite its significance, non-emergency patient transportation services are an underdeveloped aspect of health system strengthening.

Transportation systems are essential in achieving universal health coverage and should be integrated into health system planning, but this has not been prioritised in South Africa’s health system transformation programmes.^8^ Continued lack of access to effective transportation services remains a barrier to timely access to healthcare, especially for vulnerable and rural communities. Research has shown that transportation barriers can result in missed appointments, delayed treatment and suboptimal treatment adherence, which can, in turn, lead to worse health outcomes and higher healthcare expenses.^9,10^ Insufficient or inefficient PPT services may compound these problems by delaying patient transfers and decreasing service efficiency.

Transportation obstacles have been recognised globally as a barrier to healthcare. For instance, delayed access to care because of transportation barriers has been estimated to affect millions of people in high-income countries.^6,11^ While national data are scarce in SA, reports from the South African Human Rights Commission suggest that the lack of adequate or non-existent public transport services in many parts of the country severely limits access to health services, especially for the most disadvantaged.^12^ Such barriers are exacerbated in remote regions, where infrastructure and transport infrastructure are lacking, making access to timely healthcare more difficult.^13^

Access to care delays because of transport issues have been linked to poor health outcomes, including disease progression, morbidity and mortality.^14^ Inadequate PPT services have also been shown to result in increased healthcare costs and the deterioration of treatable disease into emergencies.^4^ Additionally, transport-related access issues, including lack of road infrastructure and public transport, disadvantage rural populations, particularly in their ability to access timely healthcare, perpetuating health inequalities.^15^

In SA, the National Health Insurance (NHI) model acknowledges the need for access to healthcare services, but does not provide specific mechanisms to address patient transportation issues.^16^ This represents a significant gap in policy and practice, particularly with respect to non-emergency patient transport systems. This research contributes to this area by investigating the contribution of PPT to improving access to healthcare services, and identifying current challenges and practices that may guide approaches to optimise the system and enhance health outcomes in KZN, SA.

This study describes the current practices of planned patient transportation in KZN, SA.

## Research methods and design

### Study design

A qualitative research approach using a descriptive design was employed to explore and describe the current practices of PPT in KZN, SA.

### Setting

The study was based in KwaZulu-Natal Emergency Medical Services (KZN EMS) in the northern region of KZN, SA, which consists of three districts. Emergency Medical Service King Cetshwayo district serves as a central point for the PPT services in the King Cetshwayo, Umkhanyakude and Zululand districts. Planned patient transportation services within the North Cluster of KZN are coordinated through a centralised hub based at Ngwelezana Hospital. The hub serves the King Cetshwayo, Umkhanyakude and Zululand districts and operates scheduled weekday routes to transport patients requiring specialist services at regional, tertiary and central hospitals, as well as to facilitate the repatriation of discharged patients to their base hospitals. The service is staffed by operational personnel or Prehospital Emergency Care Providers (PECPs) and PPT coordinators and utilises a mixed fleet of buses and stretcher-equipped vehicles. The hub-and-spoke model was introduced in 2010 to optimise limited transport and human resources across the three districts and improve access to specialist healthcare services. It has 14 routes on weekdays to transfer patients to higher-level hospitals and repatriate them to the base facilities. It also supports Tuberculosis (TB) and Medical Male Circumcision (MMC) programmes and provides non-emergency transport between clinics and hospitals within the parts of the district, ensuring that patients are returned to their primary healthcare facilities of referral.

### Sampling and recruitment strategy

The study population comprised PECPs and EMS management staff working within the Zululand health cluster of KZN, SA. Participants were drawn from the public EMS sub-sector operating in rural districts and included personnel with direct involvement in PPT services.

#### Recruitment of emergency medical services managers

Purposive sampling was employed to select EMS managers who possessed relevant knowledge and experience regarding PPT operations.^16^ Gatekeeper permission was obtained from the Director of KZN EMS, who facilitated access through district EMS managers. District managers informed eligible EMS managers involved in PPT services about the study and confirmed their availability for participation. Managers occupying supervisory and operational leadership positions were identified as information-rich participants as a result of their involvement in decision-making, resource allocation and oversight of PPT services within the Zululand Cluster.

#### Recruitment of prehospital emergency care providers

Prehospital emergency care providers were recruited using a maximum variation purposive sampling strategy to ensure representation across district and operational settings, while capturing a broad range of experiences within different operational contexts in the Zululand cluster.^17^ Three districts, namely Umkhanyakude, King Cetshwayo and Zululand, were selected to reflect variation in service delivery environments. EMS bases within these districts were identified, and PECPs were recruited from selected bases to participate in FGDs. Within these bases, eligible PECPs were selected based on their direct experience in PPT services and operational availability. This approach ensured representation of PECPs across different service environments within the cluster.

#### Inclusion criteria

Participants were included if they were EMS management staff or PECPs with direct involvement in PPT services within the study setting. Selection was guided by their operational experience, knowledge of PPT processes and ability to provide detailed information relevant to the study objectives.^18^

### Pilot study

A pilot study was conducted to test the semi-structured interview and focus group discussion guides for clarity, relevance and feasibility.^19^ Pilot participants were selected using the same inclusion criteria as the main study but were excluded from the final analysis. The pilot was conducted in iLembe and uMzinyathi health districts of KZN, involving three EMS managers and ten PECPs. The pilot study also confirmed the appropriateness of the questions and the overall feasibility of the study procedures.

### Data collection procedures

Following approval from the Department of Health and receipt of gatekeeper permission, preliminary and follow-up meetings were held with district EMS managers to facilitate participant recruitment and schedule suitable dates for interviews and FGDs. Participants were invited through official EMS communication channels, and their availability was confirmed through district management structures. Data collection was conducted over a 3-month period at mutually agreed times in EMS boardrooms, hospital meeting rooms and EMS stations.

Before participation, all participants received an information sheet explaining the purpose, procedures, risks and benefits of the study and subsequently provided written informed consent. Verbal consent for audio recording was also sought and recorded at the start of each interview and FGD prior to the commencement of data collection. The principal investigator conducted all semi-structured interviews and FGDs face-to-face. Eleven EMS managers participated in individual interviews. Interviews continued until no new themes or insights emerged from successive interviews. Data saturation was reached after the tenth participant, and validation was done by the eleventh participant, at which point data collection ceased.

Five FGDs were conducted with a total of 35 PECPs, with the demographic of participants shown in Table 1. The number of participants (35) was guided by focus group methodological considerations.^18^ Participants in FGDs were grouped according to their work locations to facilitate discussion of shared operational experiences related to PPT. Interviews and FGDs lasted approximately 45–60 min. The FGDs and interviews were mainly held in English; however, participants were not forced to respond only in English, but were free to express themselves in isiZulu when they felt more comfortable doing so. Where isiZulu was used, the relevant sections were translated into English during transcription to ensure accurate representation of participants’ views.

**TABLE 1:** Demographic characteristics of focus group discussion participants (*N* = 35).

Code	*n*
FGD1	4
FGD2	7
FGD3	7
FGD4	11
FGD5	6

Interview and FGD guides were developed from the study objectives and a review of relevant literature on PPT and healthcare referral systems.^18,20^ The guides shared the same core topics and questions to ensure consistency across data collection methods, although minor adaptations were made to the FGD guide to facilitate group interaction. The guides were reviewed by the research supervisors to ensure clarity and alignment with the study objectives.

Interviews and FGDs were audio-recorded with a digital recorder, and field notes were taken concurrently to capture contextual observations. Participants’ anonymity was maintained by excluding identifying information from recordings and transcripts. Audio recordings were transcribed verbatim for analysis and stored electronically in password-protected files to ensure confidentiality and data security. The researcher’s previous employment within KZN EMS aided in establishing a sense of understanding and rapport building with participants; however, reflexive awareness was maintained throughout the process of data collection to minimise potential bias.

### Data analysis

Thematic analysis was used to analyse the data, which facilitated exploration of participants’ views, experiences and understandings of the PPT system.^21^ Interview and FGD recordings were transcribed verbatim and imported into NVivo (Computer-Assisted Qualitative Data Analysis Software [CAQDAS]) to facilitate data management, coding consistency and theme development. The analysis was conducted inductively and iteratively, with codes continuously compared and refined to identify recurring patterns and meanings within the dataset.^22^ A preliminary codebook was developed to organise emerging codes, themes and subthemes, and was revised throughout the analytical process as categories were merged, refined or removed.

To ensure trustworthiness, several measures were taken. Initiating prolonged engagement with participants and conducting both semi-structured interviews and FGDs for data triangulation strengthened the level of credibility. All transcripts were verified for accuracy, and the coding process and thematic analysis were reviewed by a different member of the research team to ensure that the findings were consistent and credible. The principal investigator did the initial coding, but codes were discussed throughout the coding and development of their meanings with the research team to ensure that the themes that emerged were grounded in the data.

Reflexivity was ensured throughout the study by holding reflective notes in order to identify and minimise any potential bias that may have developed from the researcher’s prior experience in the EMS system. Structured interview guides, audit trails of analytical decisions and development of themes and ongoing oversight by the research team were used to enhance dependability and confirmability. Transferability was enhanced by the descriptions that were provided of the study setting, the participants and the results, which allowed readers to judge the transferability of the results to other contexts.

### Ethical considerations

The study was approved by the Durban University of Technology (DUT) Institutional Research Ethics Committee (IREC). The ethical clearance number is IREC 323/24. Approval to conduct the study was also obtained from the National Health Research Database (NHRD) and the KZN Department of Health (DoH).

## Results

The study included 46 participants from the Zululand Cluster of KZN EMS, comprising 11 EMS managers and 35 Prehospital Emergency Care Providers. The demographic profile of interview participants is presented in Table 2, and details of focus group discussion participants are presented in Table 1.

**TABLE 2:** Demographic profile of interview participants.

Participant	Age (Years)	Gender	Professional category	Clinical experience (Years)
P1	49	Male	EMS shift leader	31
P2	45	Male	EMS shift leader	12
P3	45	Female	EMS shift leader	11
P4	50	Male	EMS shift leader	24
P5	37	Female	EMS shift leader	14
P6	55	Male	EMS shift leader	17
P7	47	Male	EMS shift leader	15
P8	54	Male	EMS sub-district manager	11
P9	61	Male	EMS sub-district manager	35
P10	45	Male	EMS shift leader	20
P11	46	Male	EMS shift leader	21

EMS, emergency medical services.

Eight themes with their sub-themes were identified from the in-depth interviews and focus group discussions. These themes depicted the participants’ views on factors that influence PPT in KZN. Following thematic analysis, the identified themes were grouped and organised into two overarching categories as shown in Figure 1: facilitators of PPT and barriers to PPT. Given the breadth of the findings, the themes relating to barriers to PPT were reported separately, while this manuscript focuses on themes relating to facilitators of PPT. This study explored themes that reflected enablers of effective PPT, such as ‘Digital transformation in clinical practice and operational innovation’, ‘Integrated stakeholder collaboration and community engagement’, ‘Decentralised healthcare delivery’ and ‘Policy coherence’. Table 3 summarises the themes and subthemes derived from participants’ knowledge and perceptions. Themes and subthemes identified are outlined below, with exemplar quotations.

**FIGURE 1:**
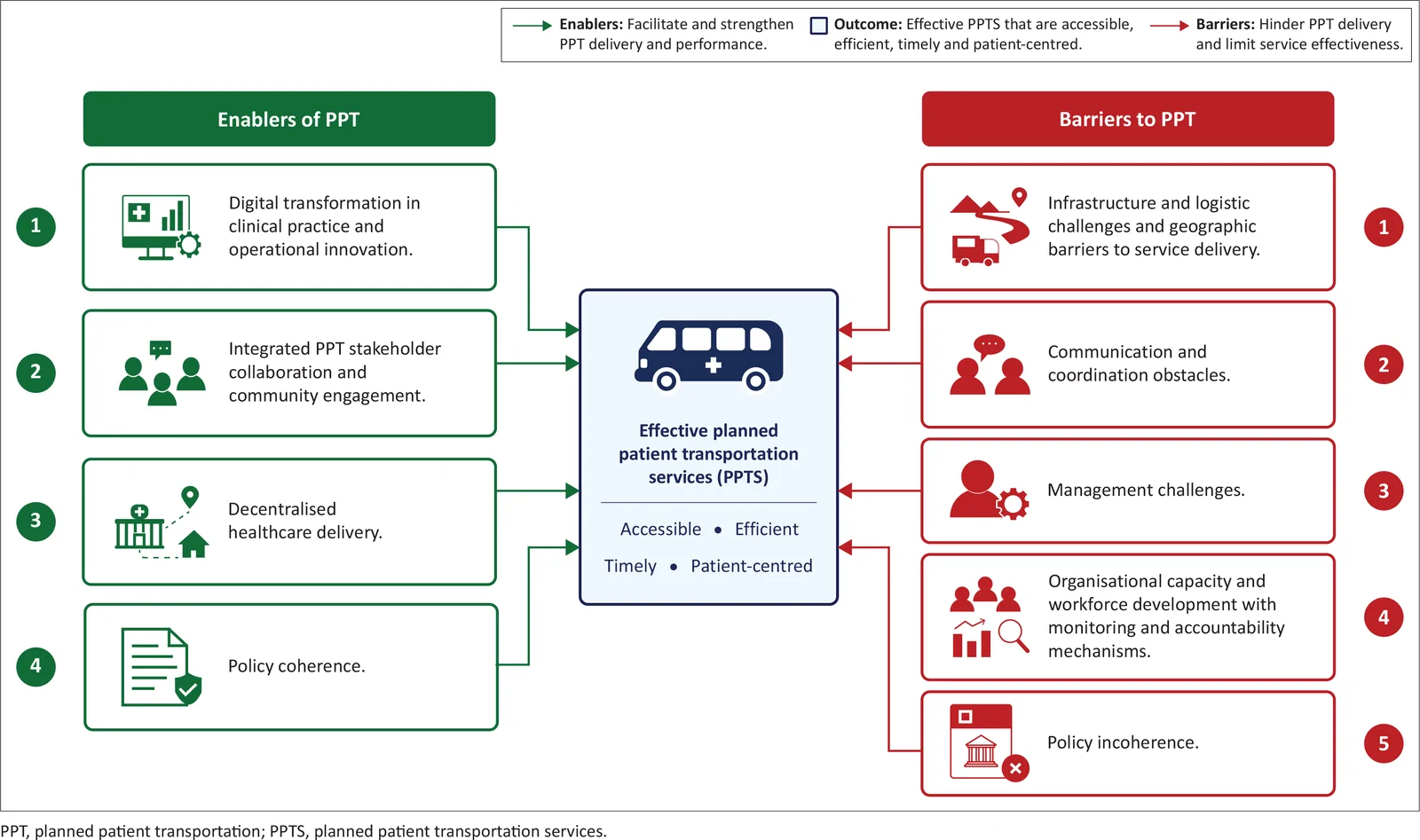
Conceptual framework of factors influencing planned patient transportation.

**TABLE 3:** Summary of themes and subthemes.

Themes	Subthemes
1. Digital transformation in clinical practice and operational innovation	1.1 Need for digitalised booking and record-keeping. 1.2 Performance monitoring and reflective practice. 1.3 Operational preparedness and structured PPT coordination.
2. Integrated stakeholder collaboration and community engagement	2.1 Collaboration and system integration between EMS and hospitals. 2.2 Feedback mechanisms from the community.
3. Decentralised healthcare delivery	3.1 Decentralised hospital-based patient transport. 3.2 Cross-district referral pressures and regional capacity constraints.
4. Policy coherence	4.1 Adherence to Standard Operating Procedures in implementation. 4.2 Resource adequacy and policy support for effective operations.

### Theme 1: Digital transformation in clinical practice and operational innovation

This theme was a cross-combination of three sub-themes, ‘Digital transformation in clinical practice and operational innovation’. This theme focused on the importance of data-driven modernisation, integration and performance monitoring for efficiency and quality improvement, and the need for innovative technological solutions and operational preparedness for strengthening PPT services. The sub-themes identified under this theme were ‘Need for digitalised booking and record-keeping’, ‘Performance monitoring and reflective practice’ and ‘Operational readiness and coordinated PPT’. Some of the other sub-themes were not included here as they were not relevant to this study.

Participants highlighted the need to transition from paper-based and phone-based booking systems and move towards digital systems to manage PPT requests. They explained that the current manual record-keeping system leads to the loss of information and inefficiencies, and the need for digital systems to enhance information management, access and continuity of services. One participant explained:

‘The entire system must go with the times … We can’t be using the old system that we’ve been using 10 years ago. Right now, we are using telephones to book patients and writing things on paper … Once that paper is lost, it’s an issue because the whole booking process must start afresh … We must keep the data digital … If things are digital, it’s easier to go back and rectify documents. The system must move with the times and embrace transformation.’ (P7)

Participants identified the need for performance monitoring and reflection to enhance the efficiency and effectiveness of PPT services. They stressed the importance of collecting information, reviewing operational reports and having an evaluation framework to monitor service performance, pinpoint problems and inform remedial strategies. One participant explained the need for job evaluation and the regular review of transport data as follows:

‘They need to come up with a job evaluation for those performing PPT services … They must look at the total number of patients transported and categorise them according to the type of illness or condition … That data should indicate how many categories of patients are transported daily, weekly and monthly, including the age of the patients. Then that information …’ (P9)

Another participant emphasised the role of the DoH’s performance management system to improve monitoring and evaluation of PPT services by saying:

‘Uhm, we have, we, the Department of Health. Actually uh, government has got such, you have heard of something called performance management and development system? Yeah, that thing is a very, is a very sensitive and effective tool, but if used properly, as said, if we appoint someone, if institutions appoint someone to, to take care of that and then doing those duties, seeing to it that PPT is effective. As, as key uh, performance areas, as the key performance areas that that’s the role as a responsibility only for that person. The focus of that, of that person will only be the smooth running on the PPT. I think that tool is sensitive and useful and efficient enough, but as long as the duties are outlined clearly to whosoever that is touched with that responsibility.’ (P10)

While another participant reported:

‘Yeah. If I implement Yes, I need to, to assess, Yeah, like, how we going, basically, like, how we look at our month end report, how we look at it, and we, here, in our district, we sit at it and we go through it in detail, yeah, we scrutinize and see you not where, where we, you know, misled, where we missed, took patients, where we took extra patients, you know, stuff like that. Maybe looking at whatever we implement in the framework, can be analysed all our staff, where we can sit as a group, obviously institutionalise it, but also not to hurt anyone’s feelings (Laughs) …’(P1, Focus group discussion 4)

Participants perceived operational preparedness and structured coordination as essential for improving the efficiency of PPT services. They emphasised the importance of early transport plans, supervision, staffing and communication with patients and facilities receiving PPT patients. One participant explained:

‘At times patients are delayed, particularly when travelling long distances such as to Durban. If the staff who transport them started early then the patients would get help early. Sometimes there is more focus on the staff working hours rather than ensuring that patients receive timely care.’ (P1, Focus group discussion 4)

Another participant added:

‘The planned patient transportation service should have a supervisor, its own vehicles and enough staff. Patients should be called early to confirm their availability to be transported and receiving hospitals should be notified to ensure that they are prepared to receive the patients.’ (P5, Focus group discussion 4)

### Theme 2: Integrated stakeholder collaboration and community engagement

Planned patient transportation stakeholder collaboration and community engagement was a prominent theme in the interviews and the FGDs’ themes. This theme emerged from the amalgamation of several related categories such as gaps in co-ordination between EMS and hospital staff, integrated communication for continuity of care, stakeholder communication and engagement for system alignment, collaborative evaluation and stakeholder feedback, and community engagement. These themes collectively emphasise the need for communication, role alignment and collaboration between various stakeholders in PPT. This section highlights some of the sub-themes that exemplify the benefits of collaboration and community engagement for PPT: ‘Collaboration and integration between EMS and hospitals’, ‘Communication and coordination frameworks in PPT operations’, ‘Stakeholder communication, engagement and accountability in PPT services’ and ‘Community feedback mechanisms’.

Participants noted that EMS and hospital collaboration and system integration are crucial to the smooth operation of PPT services. They stressed the importance of mutual cooperation, good communication and joint operation planning between EMS, hospital and receiving facilities to facilitate patient transfers and avoid delays. One participant explained:

‘We need mutual cooperation with the hospitals … If we have a breakdown or patients are left behind, hospitals must assist us when we ask for help … We also need managers that understand PPT and take responsibility. Before they go home, they must make sure that all buses are operational, and if not, make arrangements with the affected hospitals … If we can get managers who understand how PPT works, then it’s going to be fluent to work.’ (P6)

Another participant noted a lack of communication between EMS and receiving hospitals:

‘In a situation like this one we are in; communication should come first. There is a lack of communication from EMS and also between facilities, especially with the hospitals we transfer patients to. Receiving hospitals lack communication. Even among doctors, there is a gap sometimes a patient is booked as stable but marked as urgent. When you arrive there, it can take two to three hours. So, the lack of communication is another point of weakness; communication is lacking in that area.’ (P3)

The other participant added:

‘Yeah, uh, yeah. that is one of those. And another thing, which is, which is causing, uhm, which is some of the obstacles in our way, is that the time that is spent at Ngwelezana when you transport patients to clinics for those planned now, the patients, patients were discussed either with the doctors. It’s either it’s urology, orthopaedic or whatsoever eye clinic, whatsoever at Ngwelezana, Then, but then, the time that was that will be spent by the paramedics and the vehicle. Yeah, inside, inside hospital now, more than three to four, five hours, but yet the patient was planned. In other words, then there is the receiving facility was aware that the patient is coming. Yeah.’ (P10)

Participants emphasised the need for effective feedback channels to and from the community to PPT services. They explained that patients can feel uncomfortable sharing concerns about transportation, and more involvement with community groups may help convey community feedback to increase the responsiveness of services. One participant explained:

‘The patients mustn’t be afraid to tell the doctors that I cannot climb in the bus. So, they are afraid, they are not involved. So, involvement is not that. I’m not sure whether they are afraid of the doctors or the nurses, or they’re afraid that they’re not going to be able to go to the hospital. I’m not sure, but they allow everything even if it’s, it’s not, it’s not okay. But they allow everything even if it’s wrong. Yeah, they allow everything. They, they say yes, yes, yes. Everything is yes. There is no, no to them …’(P1, Focus group discussion 3)

Another participant recommended liaising with community structures to get feedback on community perspectives:

‘I think at the moment, we have some local government structures where, if you get to, to their meetings that involve the ward councillors and staff, maybe our managers, if they can liaise with the local councillors when they have the community meetings, and they must be also involved in those meetings just to find out, what is the, what is the community feeling about the service that you render, yeah, so maybe that can help to get the, what, what the, the opinion of the community is? if you go to the local meetings, but that’s the responsibility of the managers, yeah, but with us, they must, they mustn’t leave us behind; two or three of us, the, the staff on the ground because, some questions they cannot answer them. We deal with, with the people, with the patients on daily basis, at hand …’ (P1, Focus group discussion 1)

### Theme 3: Decentralised healthcare delivery

Participants defined decentralised hospital-based patient transport as a model that enables hospitals (including sub-district facilities) to have responsibility for their own patient transport. They explained that a decentralised approach to transport could lead to increased capacity, reduced travelling distances and efficiency in patient transport within the district. One participant explained:

‘So basically, yeah, it was basically they were doing their own transportation, yeah, but not in such big numbers. And we look, that’s why they look at it. If you look at it around Mosvold will take their own patients. Manguzi take their own patients. So Itshelejuba take the patients, So, how many vehicles they were using to transport minimum patients, because the maximum they could take for 16 per hospital.’ (P1)

Participants also explained ways to improve decentralised healthcare services, such as establishing regional hospitals in the district, would assist with reducing cross-district referrals and the strain on PPT services:

‘Yeah. And if they, we can have a regional hospital within the district. It can….’ (P9)

Participants emphasised that inter-district referrals place a heavy burden on regional hospitals, leading to overcrowding and delays in patient care and transport. One participant explained:

‘What I can say, it’s uh, there is what we call the, it’s the plan that we, UMkhanyakude need to have the Regional Hospital. Oh, yeah. Uh, so that Regional Hospital will assist those far away, those four hospital that is within the district. Uh, but if we are looking at Ngwelezana, uh, receiving patients from Zululand and receiving patients from UMkhanyakude and I’m not sure as how many, the referrals district hospital within King Cetshwayo. Yeah. So, it looks like Ngwelezana end up being overloaded, yes, because, like five hospitals within UMkhanyakude.’ (P9)

Another participant noted how increased patient volumes lead to delays in patient care and transport turnaround times:

‘I’ll repeat one and the same thing. That’s the number of patients as we do transport, it’s higher, yes. Two, Uh, the delay in our hospitals during patient transport. They don’t get helped quickly, but I won’t blame them, because it’s the number of patients, which means they’re treating, but it’s not quicker. They delay in a hospital when transporting patients, because ambulance used to leave early in the mornings, comes late in the afternoon.’ (P4)

### Theme 4: Policy coherence

The importance of adhering to Standard Operating Procedures (SOPs) in the delivery of PPT services was highlighted. Participants noted that SOPs offer a guide to decision-making, enabling consistency and adherence to regulatory guidelines. One participant explained:

‘So, in most cases, every department or component, they’ve got their processes, you know, for and procedures to, to implement anything and their protocols. Unfortunately, fortunately in EMS, we are, everything that we are doing must be in line with our SOP, with our Department of Health Standard Operational Procedures. So, we first looking at that on those processes that we are going to meet, whatever that we want to implement, so that we won’t have a problem that we have violated someone’s right or something.’ (P7)

Sufficient resources and policy support were identified by participants as essential for effective PPT services. Participants noted that having adequate and sustainable resources, in line with policy frameworks and service needs, is critical to effective service delivery. One participant explained:

‘I think to this point, it can be uh, getting the resources and including the policy, framework uh, including the manpower. I think the system will run smoothly and the right resources, these three needs, because that is the main point, because some of these resources are not suitable for the needs of the district. Just a vehicle that can come, three months down the line is gone, and some of them, they’re gone forever, and they stay in the workshop until they’ve been boarded. So, yes …’ (P11)

## Discussion

This study highlighted existing practices that may be used to guide approaches to improve PPT in KZN, SA. The findings highlighted that PPT plays a pivotal role in ensuring healthcare access, especially in tiered health systems where patients move between primary, district, regional and tertiary levels of care. However, the effectiveness of PPT is influenced by multiple interacting factors, including referral coordination, communication, resource availability, organisational capacity, governance and policy implementation.^3,23,24^ These collectively have important implications for District Health System performance, continuity of care and equitable access to healthcare services within the KZN EMS context and similar District Health System settings.^25^

The first theme, digital transformation in clinical practice and operational innovation, highlighted the importance of digitalised booking systems, electronic record-keeping, performance monitoring and structured coordination of PPT services. Participants recognised that data utilisation, monitoring and evaluation processes, and operational preparedness are essential for improving efficiency and accountability. Evidence suggests that fragmented data systems within public healthcare settings impede service delivery and limit decision-making capacity.^26^ Consistent with international literature, participants recognised the potential value of electronic referral systems, transport booking platforms, patient tracking systems and real-time fleet management technologies. These digital health interventions may strengthen coordination, improve accountability, optimise resource utilisation and reduce delays associated with poor communication.^27^ The results therefore indicate that digital health systems could be important enablers in supporting referral pathway strengthening and continuity of care in the context of the EMS District Health Systems in KZN.^6,28,29^

The second theme focused on collaboration between EMS, healthcare facilities and community stakeholders through integrated stakeholder collaboration and community engagement. Good communication and teamwork between healthcare facilities and EMS staff were cited as key facilitators of PPT. The improvement of referral systems and timely patient transfers across levels of care is a crucial aspect of communication. Similar findings have been reported in South African and international studies, where ineffective communication contributed to delays in accessing appropriate care and disrupted referral processes.^29,30^ Within the KZN EMS District Health System context, coordinated communication supports service integration and continuity of care by facilitating seamless patient movement between facilities. Participants also highlighted the value of stakeholder engagement and community feedback mechanisms in strengthening accountability and improving service responsiveness.^29^ These findings suggest that successful PPT depends not only on infrastructure and resources, but also on the quality of relationships and coordination between organisations involved in patient care.

Efficient PPT systems within the study setting support referral coordination and enable timely access to specialised services. Consistent with findings reported by Dixon et al.^31^ and other studies, coordinated transport and referral systems can reduce geographical and service inequalities, particularly within resource-constrained settings. However, the present findings extend beyond the availability of transport by demonstrating the importance of system-level coordination in maintaining continuity of care.^29,31,32^ This is particularly relevant for patients with chronic conditions and those requiring repeated referrals between healthcare facilities.

The third theme, decentralised healthcare delivery, revealed both opportunities and challenges for PPT. Participants reported that decentralised services improve access by bringing healthcare closer to communities. This aligns with primary healthcare principles that promote accessibility, local accountability, and responsiveness, and was reflected in participants’ experiences within the study setting. However, participants also reported challenges related to inter-district referrals and pressures on district and regional hospitals.^13,29^ Similar challenges have been documented in other studies conducted by Neely and Ponshunmugam, Sapkota et al., and Birru et al. in SA and low- and middle-income countries (LMICs), where decentralisation may improve access but can also create fragmentation when referral systems, governance structures, infrastructure and resources are insufficiently aligned.^13,33,34^ These findings indicated that the advantages of decentralisation are linked to strong referral networks, the effectiveness of communication systems and the coordination of district-level planning. From a family medicine perspective, decentralisation requires functional systems that ensure continuity and coordination of care across the healthcare continuum.

The fourth theme, policy coherence, highlighted the importance of adherence to SOPs, policies, guidelines and performance benchmarks in facilitating effective PPT. Collaborative policy frameworks improve health system coordination, accountability and communication at all levels.^24,29^ This is consistent with local and international evidence that highlighted the importance of governance frameworks in supporting integrated healthcare delivery. Participants further identified adequate vehicle availability and staffing as critical enablers of PPT. Similar to previous South African studies by Mubaiwa and Mathews et al., transport resources were recognised as necessary for reducing delays, missed appointments and disruptions in care.^8,24^ However, unlike studies that primarily attribute transportation performance to resource availability,^32^ participants in the present study also emphasised organisational factors, leadership accountability and effective policy implementation as important determinants of transportation effectiveness. This implies that greater governance over coordinated and equitable service provision, in addition to more resources, might be needed to improve PPT in the study setting.

Collectively, these findings highlight that effective PPT in the KZN EMS study area is not only about the availability of transport, but also about the interaction between digital innovation, collaboration among stakeholders, decentralised service delivery, resource availability and coherent policy implementation. Consistent with South African and international literature, transport system performance is influenced not only by resources, but also by organisational, technological and governance factors. Strengthening these interconnected components could contribute to better coordination of referrals, continuity of care and equitable access to healthcare services.

### Implications

The findings suggest that coordinated health system processes, such as high-quality referral pathways, effective communication, digital health integrations, organisational leadership and policy frameworks, are important for effective PPT. Reliable PPT enables patients to access appropriate levels of care in a timely manner, thereby supporting continuity of care and reducing disruptions in treatment. For family physicians and primary healthcare teams, functional transportation and referral systems are essential for coordinating patient-centred care across different levels of the health system. Strengthening these facilitators may enhance healthcare access, improve service integration and support the delivery of comprehensive primary healthcare in similar District Health System environments.

### Recommendations

A systematic approach to maximise PPT systems is required, which includes the digital transformation of systems, improved stakeholder engagement and coordination, decentralised service provision and referral networks. Organisational capacity building, staff training and resourcing capacity building are important for sustainability. In addition, strengthening policy coherence through standardised procedures and robust monitoring systems, alongside incorporating community feedback, is critical to ensuring responsive and patient-centred services.

### Strengths and limitations of the study

A major strength of this study was the use of a qualitative design to allow the whole range of participants’ experiences, perceptions and recommendations about PPT in the KZN EMS context to be explored. Both semi-structured interviews and focus group discussions were used to achieve methodological triangulation and rich, detailed accounts from various stakeholder groups. Prolonged engagement with participants during data collection, combined with iterative discussions, allowed for an exploration of key issues. Data collection continued until data saturation and the themes were sufficient to represent participants’ perspectives. Furthermore, the use of a systematic inductive thematic analysis enabled the identification and interpretation of patterns across the dataset, contributing to a nuanced understanding of factors influencing PPT.

The study also had several limitations. Data collection was constrained by the operational rosters of the PECPs, which limited opportunities for participant engagement and made access to participants during their working shifts challenging. In addition, the findings were based on participants’ experiences and perceptions and could have been subject to recall or social desirability biases. The findings may not be directly transferable to other provinces or healthcare settings with different organisational and operational contexts, as the study was conducted in Northern KZN EMS. However, it is possible that some of the identified facilitators may be relevant to similar EMS and PPT settings, and potentially to other provinces or broader healthcare contexts facing comparable resource, referral and coordination challenges. Nevertheless, the diversity of participants’ professions and the application of a variety of data collection approaches provided credibility and depth to the findings.

## Conclusion

Planned patient transportation plays a crucial role in the District Health System in terms of access and continuity of care for patients. The findings suggest that strengthening referrals, standardising policies and operating procedures, improving communication between stakeholders, investing in digital health and data management systems and allocating sufficient resources are key priorities for improving PPT. These findings have significant policy implications for primary healthcare and family medicine because successful PPT can facilitate timely referrals, patient-centred care and equitable access to health services. The Department of Health should prioritise integrated evidence – informed intervention to enhance the governance, coordination, and service delivery to ensure the sustainability, effectiveness and responsiveness of PPT services in KZN and similar settings.

## References

[1] KwaZulu-Natal Department of Health. Annual report 2011/12 [homepage on the Internet]. Pietermaritzburg: KZN Department of Health; 2012 [cited 2026 Jun 26]. Available from: https://www2.kznhealth.gov.za/2011-2012-Annual-Report.pdf

[2] Baghbanian A, Torkfar G. Economics and resourcing of complex healthcare systems. Aust Health Rev. 2019;56(2):113–116. 10.1071/AH11041 22958896

[3] Nguru K, Ireri L. Challenges influencing proper implementation of quality health care referral system in Kaloleni Sub-county, Kilifi County in Kenya. Int Emerg Nurs. 2022;62:101169. 10.1016/j.ienj.2022.101169 35483211

[4] Andritsos D, Aflaki S. Competition and the operational performance of hospitals: The role of hospital objectives. Prod Oper Manag. 2019;68(2):16–20.

[5] Ann S. Motivating senior employees in the hospitality industry. Int J Contem Hosp Manag. 2020;32(1):20–100. 10.1108/IJCHM-08-2018-0685

[6] Give C, Ndima S, Steege R, et al. Strengthening referral systems in community health programs: A qualitative study in two rural districts of Maputo Province, Mozambique. BMC Health Serv Res. 2019;19(1):263. 10.1186/s12913-019-4076-3 PMC6489304 31035983

[7] Stewart J, Wolvaardt G. Hospital management and health policy – A South African perspective. J Hosp Manag Health Policy. 2019;3:14. 10.21037/jhmhp.2019.06.01

[8] Mubaiwa T. An empirical investigation on the provision of non-emergency patient transportation services in South Africa. Cape Town: University of Cape Town; 2018.

[9] Gray A, Vawda Y. Heath policy and legislation. S Afr Health Rev. 2021;78(1):89–91.

[10] Androniceanu A. Transparency in public administration as a challenge for a good democratic governance. J Public Manag. 2021;56(2):114–118.

[11] Ge B, Anderson G. More detailed understanding of factors associated with hospital profitability. Health Aff. 2020;67(3):90–91. 10.1377/hlthaff.2015.1193 27140996

[12] Abrahams G, Thani X, Kahn S. South African public primary healthcare services and challenges. Adm Publica. 2022;30(2):63–85.

[13] Neely AH, Ponshunmugam A. A qualitative approach to examining health care access in rural South Africa. Soc Sci Med. 2019;230:214–221. 10.1016/j.socscimed.2019.04.025 31030012

[14] Solomon EM, Wing H, Steiner JF, Gottlieb LM. Impact of transportation interventions on health care outcomes: A systematic review. Med Care. 2020;58(4):384–391. 10.1097/MLR.0000000000001292 31985588

[15] Alamanou D, Brokalaki H. Intrahospital transport policies: The contribution of the nurse. Health Sci J. 2014:113–117.

[16] Weimann E, Stuttaford MC. Consumers’ perspectives on National Health Insurance in South Africa: Using a mobile health approach. JMIR mHealth uHealth. 2014;2(4):e3533. 10.2196/mhealth.3533 PMC4259968 25351980

[17] Berndt AE. Sampling methods. J Hum Lact. 2020;36(2):224–226. 10.1177/0890334420906850 32155099

[18] Creswell JW, Creswell JD. Research design: Qualitative, quantitative, and mixed methods approaches. 5th ed. Thousand Oaks, CA: SAGE Publications; 2017.

[19] Aziz A, Khan N. The potential uses of pilot study in qualitative research. J Res Rev Soc Sci Pak. 2020;3(1):750–767.

[20] Ahmad S, Wasim S, Irfan S, Gogoi S, Srivastava A, Farheen Z. Qualitative v/s. quantitative research – A summarized review. Population. 2019;1(2):2828–2832.

[21] Levitt HM, Bamberg M, Creswell JW, Frost DM, Josselson R, Suárez-Orozco C. Journal article reporting standards for qualitative primary, qualitative meta-analytic, and mixed methods research in psychology: The APA publications and communications board task force report. Am Psychol. 2018;73(1):26. 10.1037/amp0000151 29345485

[22] Patton MQ. Enhancing the quality and credibility of qualitative analysis. Health Serv Res. 1999;34(5 Pt 2):1189. PMC1089059 10591279

[23] Maphumulo WT, Bhengu BR. Challenges of quality improvement in the healthcare of South Africa post-apartheid: A critical review. Curationis. 2019;42(1):1–9. 10.4102/curationis.v42i1.1901 PMC6556866 31170800

[24] Mathews C, Goga A, Loveday M, et al. Moving towards universal health coverage: Strengthening the evidence ecosystem for the South African health system. S Afr Med J. 2019;109(11b):8–14. 10.7196/SAMJ.2019.v109i11b.14245 32252861

[25] Fusheini A, Eyles J. Achieving universal health coverage in South Africa through a district health system approach: Conflicting ideologies of health care provision. BMC Health Serv Res. 2016;16(1):558. 10.1186/s12913-016-1797-4 PMC5054620 27717353

[26] Achieng M, Ruhode E. EHealth technologies integration with healthcare work activities in public hospitals: A critical realist perspective. arXiv preprint arXiv:210809734. 2021.

[27] Abernethy A, Adams L, Barrett M, et al. The promise of digital health: then, now, and the future. NAM Perspect. 2022;2022:1–24. 10.31478/202206e PMC9499383 36177208

[28] Rios-Zertuche D, Gonzalez-Marmol A, Millán-Velasco F, Schwarzbauer K, Tristao I. Implementing electronic decision-support tools to strengthen healthcare network data-driven decision-making. Arch Public Health. 2020;78(1):33. 10.1186/s13690-020-00413-2 PMC7301503 32566223

[29] Matolengwe A, Murray D, Okafor UB. The challenges of implementing a health referral system in South Africa: A qualitative study. Risk Manag Healthc Policy. 2024;17:855–864. 10.2147/RMHP.S450998 PMC11017115 38623578

[30] Pittalis C, Brugha R, Gajewski J. Surgical referral systems in low-and middle-income countries: A review of the evidence. PLoS One. 2019;14(9):e0223328. 10.1371/journal.pone.0223328 PMC6764741 31560716

[31] Dixon J, Burkholder T, Pigoga J, et al. Using the South African triage scale for prehospital triage: A qualitative study. BMC Emerg Med. 2021;21(1):125. 10.1186/s12873-021-00522-3 PMC8556887 34715794

[32] Howard I, Cameron P, Wallis L, Castrén M, Lindström V. Understanding quality systems in the South African prehospital emergency medical services: A multiple exploratory case study. BMJ Open Qual. 2020;9(2):e000946. 10.1136/bmjoq-2020-000946 PMC7247383 32439739

[33] Sapkota S, Dhakal A, Rushton S, et al. The impact of decentralisation on health systems: A systematic review of reviews. BMJ Glob Health. 2023;8(12):e013317. 10.1136/bmjgh-2023-013317 PMC10749071 38135299

[34] Birru E, Ndayizigiye M, Wanje G, et al. Healthcare workers’ views on decentralized primary health care management in Lesotho: A qualitative study. BMC Health Serv Res. 2024;24(1):801. 10.1186/s12913-024-11279-3 PMC11241925 38992665

